# Micro-computed tomography-based phenotypic approaches in embryology: procedural artifacts on assessments of embryonic craniofacial growth and development

**DOI:** 10.1186/1471-213X-10-18

**Published:** 2010-02-17

**Authors:** Eric J Schmidt, Trish E Parsons, Heather A Jamniczky, Julian Gitelman, Cvett Trpkov, Julia C Boughner, C Cairine Logan, Christoph W Sensen, Benedikt Hallgrímsson

**Affiliations:** 1 Department of Cell Biology & Anatomy, The McCaig Bone and Joint Institute, and the Alberta Children's Hospital Institute for Child and Maternal Health, University of Calgary, Calgary, AB, Canada; 2 Biological Anthropology Graduate Program, University of Calgary, Calgary, AB, Canada; 3 Department of Biology, McGill University, Montreal, QC, Canada; 4 Bachelor of Health Sciences, University of Calgary, Calgary, AB, Canada; 5 Department of Biochemistry and Molecular Biology, Sun Centre for Excellence in Visual Genomics, University of Calgary, Calgary, AB, Canada

## Abstract

**Background:**

Growing demand for three dimensional (3D) digital images of embryos for purposes of phenotypic assessment drives implementation of new histological and imaging techniques. Among these micro-computed tomography (μCT) has recently been utilized as an effective and practical method for generating images at resolutions permitting 3D quantitative analysis of gross morphological attributes of developing tissues and organs in embryonic mice. However, histological processing in preparation for μCT scanning induces changes in organ size and shape. Establishing normative expectations for experimentally induced changes in size and shape will be an important feature of 3D μCT-based phenotypic assessments, especially if quantifying differences in the values of those parameters between comparison sets of developing embryos is a primary aim. Toward that end, we assessed the nature and degree of morphological artifacts attending μCT scanning following use of common fixatives, using a two dimensional (2D) landmark geometric morphometric approach to track the accumulation of distortions affecting the embryonic head from the native, uterine state through to fixation and subsequent scanning.

**Results:**

Bouin's fixation reduced average centroid sizes of embryonic mouse crania by approximately 30% and substantially altered the morphometric shape, as measured by the shift in Procrustes distance, from the unfixed state, after the data were normalized for naturally occurring shape variation. Subsequent μCT scanning produced negligible changes in size but did appear to reduce or even reverse fixation-induced random shape changes. Mixtures of paraformaldehyde + glutaraldehyde reduced average centroid sizes by 2-3%. Changes in craniofacial shape progressively increased post-fixation.

**Conclusions:**

The degree to which artifacts are introduced in the generation of random craniofacial shape variation relates to the degree of specimen dehydration during the initial fixation. Fixation methods that better maintain original craniofacial dimensions at reduced levels of dehydration and tissue shrinkage lead to the progressive accumulation of random shape variation during handling and data acquisition. In general, to the degree that embryonic organ size and shape factor into μCT-based phenotypic assessments, procedurally induced artifacts associated with fixation and scanning will influence results. Experimental designs will need to address these significant effects, either by employing alternative methods that minimize artifacts in the region of focus or in the interpretation of statistical patterns.

## Background

As powerful as routine histological methods continue to be, the demand for increasingly precise evaluations of the morphogenetic processes initiating and elaborating embryonic form has motivated applications of new technologies to the problem of imaging embryos in 3D at high spatial resolution. Various versions of "episcopic" techniques [1], including Episcopic Fluorescence Image Capturing [2] and High Resolution Episcopic Microscopy [3], generate 3D images recording details approaching histological resolutions-permitting visualization of molecular expression patterns and the distribution of cell types-embedded in the larger and natural context of an individual specimen's gross anatomical form. Their success in large part derives from successively sectioning and digitally photographing block faces containing histologically prepared specimens, a process obviating laborious external marker congruence-based methods [4-6], in addition to avoiding significant sectioning and mounting distortion artifacts inherent with traditional glass slide-mounting. Optical Projection Tomography [7,8] produces similar data non-destructively, though is limited to optically clear specimens. Both sorts of high resolution 3D visualizations are currently employed as tools in research settings: for example, see [9] for use of an episcopic approach toward detailed phenotypic assessment of heart development in mutant mice, and [10] for an analysis coupling Optical Projection Tomography to traditional methods of gene expression analysis in the developing chick limb bud.

Other non-destructive methods such as micro-MRI [11] and μCT [12-18] have been adapted for the 3D visualization of embryonic morphology. Though these methods lack the ability to readily capture concomitant molecular expression data, they possess a strong potential for high-throughput experimental designs focused on gross anatomical form [11-13]. As such, μCT can play a prominent role in quantitative studies of organismal growth and development.

**Figure 1:**
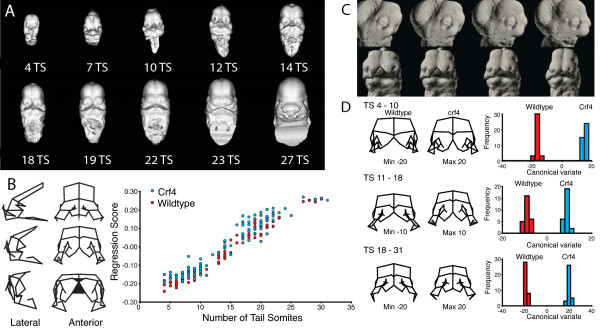
**A, Ontogenetic series of μCT scans showing the range of shape and size variation from E9.5-12**. **B**, Regression of total shape variation on tail-somite stage (TS) and wireframe deformation showing the corresponding shape trajectory. **C**, Morphing of a μCT surface render along the same shape trajectory (size constant). **D**, Comparison of the two groups at three standardized stages. Crf4 mice have a mutation on a C57BL/6J background that produces a complex set of craniofacial changes [15].

Figure 1 shows an example of μCT-based quantitative analysis of craniofacial shape variation during mouse embryonic development. This analysis employs geometric morphometrics [20,21], which is a body of methods dedicated to the quantitative analysis of shape. Analyses of this kind allow the systematic quantitative assessment of the influence of genetic factors on embryonic growth and morphogenesis, allow for the statistical assessment of differences among genotypes or treatment groups, and also contain methods for quantifying and, if desired, correcting for complex shape transformations such as those that occur during morphogenesis. Figure 1D shows an example of this, where two genotypes were compared and shapes standardized to specific developmental stages by regressing shape on tail-somite stage (Figure 1B). These methods are especially powerful if such data can be obtained from the same individual embryos that are then subsequently processed for molecular assays. This will allow investigators to correlate morphometric shape variation with molecular data, such as the expression of particular genes or density distribution of some immunohistochemical marker using individual embryos. The ability to relate directly quantitative assessment of morphogenesis with assays of molecular determinants offers an important new avenue for querying the mechanistic basis of development and dysmorphology.

There are several technical hurdles to realizing this vision of integrating morphometric and molecular data in the study of embryonic development. Fixation is a necessary measure in order to check natural degradative processes (e.g., autolysis, bacterial decay, and diffusion). The particular choice of fixative often depends on which particular histological feature a researcher wishes to demonstrate, though at the expense of fixation-induced alterations and artifacts, which are often quite significant [22-24]. Since the intent of μCT scanning embryos is, in part, to quantify morphological variation, including morphometric shape variation (e.g., [13,15]), a quantitative analysis of fixation-induced deformations is required to guide the choice of fixation method and to interpret the morphometric results of such studies. After initial fixation, additional measures in preparation for μCT scanning potentially serve as additional sources of gross morphological distortions. If fixation- and scanning-induced deformations are systematic, there is a potential to statistically adjust for them, enabling more refined quantification of the morphological variation of interest. If the magnitudes and directions of distortions are known, it may also be possible to compare samples processed with different methods.

Since accurate quantification of 3D shape variation in unfixed embryos is not possible with current methods (but see [25] for a discussion on developments in the area of *in utero *ultrasound imaging), we used two dimensional (2D) geometric morphometrics to characterize the patterns of size and shape variability in the embryonic head prior to fixation, post-fixation, and after μCT scanning. The latter set of measurements served as a proxy for the effects of scanning, plus any additional effect of re-immersing specimens in an aqueous environment (see Methods under digital photography). Direct 2D assessment of μCT-generated, 3D volumetric images was not performed. Our analysis is limited to a comparison of commonly used fixatives, Bouin's solution and two different mixtures of formaldehyde and glutaraldehyde: 4% formaldehyde + 1% glutaraldehyde, and 4% formaldehyde + 5% glutaraldehyde. For a review of multiple fixation methods including methods to improve contrast of embryonic (and unmineralized) tissues useful for μCT-scanning embryos, see [17,18].

## Results

### Scan Qualities

**Figure 2:**
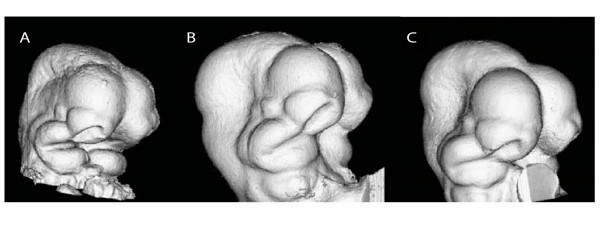
**Identically scaled renderings representing 3D μCT scans of embryos subjected to different fixation and scanning procedures**. **A**, 4% formaldehyde and secondary Bouin's fixation. **B**, 4% formaldehyde + 1% glutaraldehyde with Iothalamate meglumine used as a contrast agent. **C**, 4% formaldehyde + 5% glutaraldehyde plus contrast agent. All embryos are shown in right 3/4 inferolateral view.

Typical μCT renderings are represented by the embryos depicted in Figure 2A-C. The combination of aldehyde fixation and Cysto Conray^® ^yields images (Figure 2B-C) with a surface quality comparable to embryos fixed with Bouin's solution (Figure 2A). Bouin's-fixed embryos appear have a rougher surface texture compared to those fixed with either glutaraldehyde mixture (Figure 2B-C). The identically scaled images of Bouin's fixed embryos appeared generally smaller compared to those fixed with glutaraldehyde mixtures, but a comparison of μCT images only cannot indicate the relative roles of fixation-induced tissue shrinkage or swelling.

### Repeatability of Landmarks

**Figure 3:**
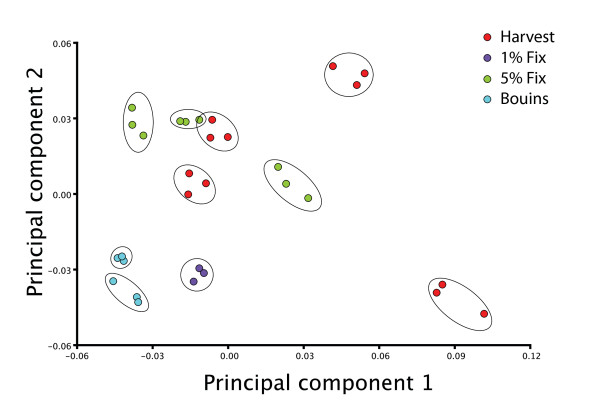
**PCA of measurement error subsample**. Each set of three repeated trials is circled, showing the distribution of among trial variation (measurement error) compared to among-individual variation in the measurement error samples.

Analysis of variance of the repeated trials of 10 embryos revealed that measurement error accounts for 10.9% of the total shape variation (relative landmark position) in a fairly homogenous sample. As shown in Figure 3, the differences between repeated trials of individuals are very small compared to the differences among individuals.

### Shape Analysis of Unstandardized Shape Data

**Figure 4:**
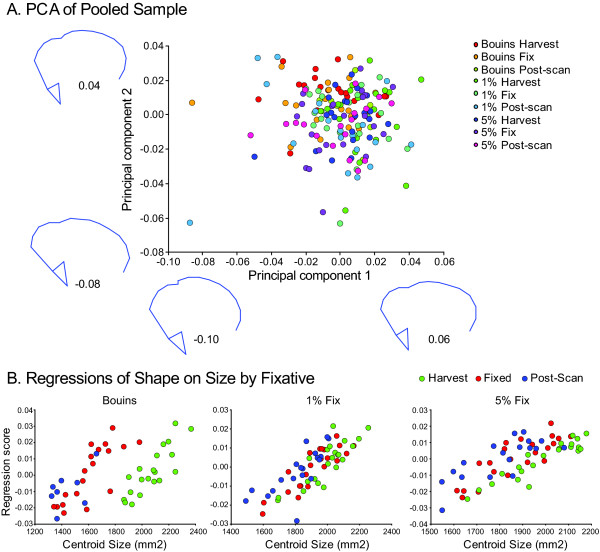
**A, PCA results for the raw Procrustes transformed data by fixation method and treatment step**. Wireframes as in Figure 1A. **B**, Regressions of shape on craniofacial (centroid) size showing variable effects of treatments within experimental groups (experimental groups left to right: Bouin's, 1% glutaraldehyde, 5% glutaraldehyde).

PCA results for the raw Procrustes transformed data by fixation method and treatment steps are shown in Figure 4A. Wireframe distortions along PC1 and PC2 indicate the presence of coherent, systematic variation within the raw dataset. Regressions of shape on craniofacial (centroid) size show variable effects of treatments within experimental groups (Figure 4B). The positive slopes of the regression lines for each of the three experimental groups demonstrate an ontogenetic component of variation in which shape change scales with size, a result not unexpected. Interestingly, unlike in either glutaraldehyde experimental group, there is within the Bouin's experimental group a conspicuous shift in y-intercept values between regression lines representing scaling effects associated with harvesting and with fixation and μCT scanning. This possibly indicates that in addition to any naturally occurring allometric variation, Bouin's fixation dramatically adds an additional source of variation. Since slopes representing the effects of fixation and post-scanning are shifted leftward, substantial craniofacial shrinkage associated with fixation is likely occurring. The trends within either glutaraldehyde experimental group, though expected, are not as apparent.

### Effects on Craniofacial Size

#### Bouin's Fixation Group

**Figure 5:**
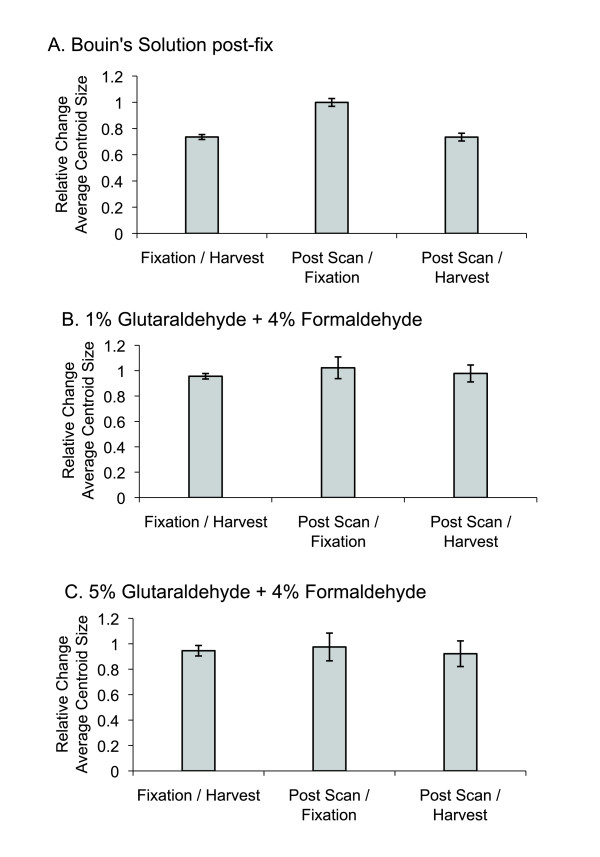
**Ratio of centroid sizes at each processing step to size at harvest or size at the previous step**. P-values provided in the text. Error bars show one standard deviation.

A cursory visual examination of 3D μCT renderings indicated that Bouin's fixation was associated with a relatively smaller craniofacial size in comparison to embryos fixed in either glutaraldehyde mixture. Centroid size analysis showed that initial overnight fixation with 4% formaldehyde followed by 3.5 hours of secondary fixation in Bouin's solution reduced average centroid size to 73.5% of the average centroid size prior to fixation (Figure 5A). The effect of μCT scanning and re-immersion into PBS for photography did little to further alter average centroid size. Average centroid size after scanning was measured to be 73.5% of average value measured pre-fixation. Average centroid size changed little between fixation and scanning, indicating that embryo shrinkage occurs mostly if not entirely during fixation as opposed to during μCT scanning.

#### 1% Glutaraldehyde Fixation Group

Overnight fixation in 4% formaldehyde + 1% glutaraldehyde reduced this group's average centroid size to 95.6% of the initial measurement (Figure 5B). Average centroid size changes little between fixation and scanning. The net effect of μCT and re-immersion in PBS for photography was to reduce the average centroid size to 97.8% the original value.

#### 5% Glutaraldehyde Fixation Group

Overnight fixation in 4% formaldehyde + 5% glutaraldehyde reduced average centroid size to 94.5% of its original value measured prior to fixation (Figure 5C). Average centroid size was reduced after μCT scanning and PBS re-immersion to 97.5% of the value measured after fixation. Compared to average centroid size pre-fixation, final average centroid size was reduced to 92.2% of initial measure.

### Effects on Craniofacial Shape: Analysis of Standardized Dataset

**Figure 6:**
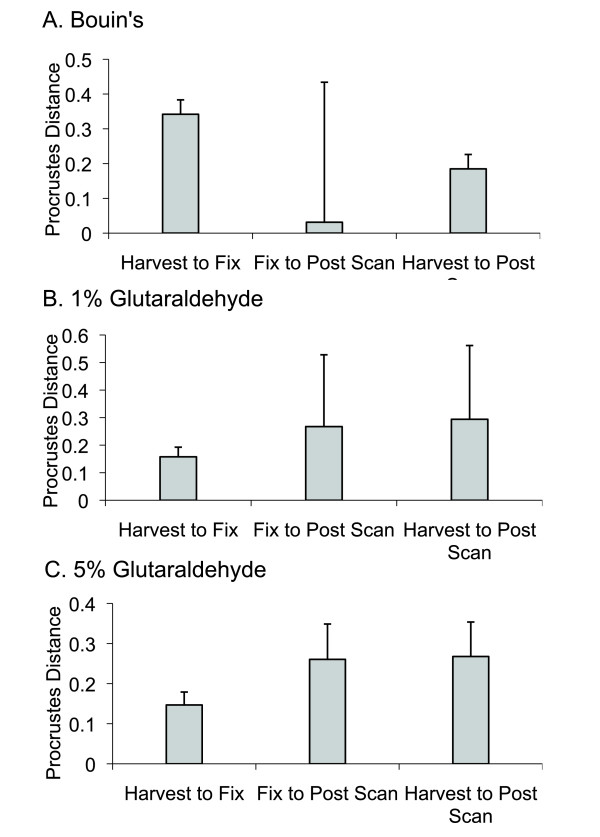
**Shape distortion, quantified by the Procrustes distance, by processing step for each fixation method**. The error bars show one standard deviation distributions around the mean shape change. The large standard deviation in the Bouin's Fix to Post Scan step is largely due to a single individual. Error bars show one standard deviation.

Alterations in craniofacial shape are associated with fixation and μCT scanning, as we detected substantial changes in standardized landmark configuration values between treatments within each fixation group (Figure 6). Within the Bouin's experimental group, the most substantial shift in Procrustes distance was associated with fixation (Figure 6A), whereas deviations in Procrustes distances more steadily accumulated during sample processing within both glutaraldehyde experimental groups (Figures 6B-C).

**Figure 7:**
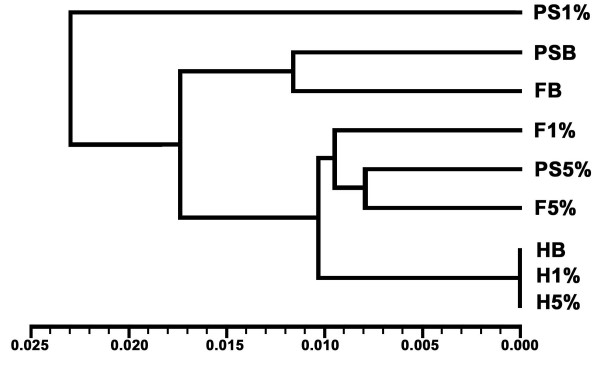
**UPGMA (Unweighted Pair Grouping using Mathematical Averaging) dendrogram derived from Procrustes distance matrix of normalized pooled-harvest sample shape data (Table 1)**. **HB, H1%, H5%**, harvested embryos of Bouin's, 1% glutaraldehyde, 5% glutaraldehyde experimental groups, respectively. **FB, F1%, F5%**, fixed embryos of each respective experimental group. **PSB, PS1%, PS5% **post-scanned embryos of each respective experimental group.

UPGMA cluster analysis (Figure 7) was applied to a matrix of tail-somite-standardized Procrustes distances and further standardized to the average landmark configuration of harvested embryos (Tables 1 and 2). The second standardization step removes between individual shape variation, leaving only treatment-induced, or "artifact" variation in the dataset. The distances separating fixation and post-scan treatments with in the Bouin's and 5% glutaraldehyde experimental groups were comparatively small in the face of the distances separating treatments within the 1% glutaraldehyde group. The 5% glutaraldehyde experimental group demonstrated the least amount of treatment induced shape distortion.

**Table 1:** Procrustes distance and P-value matrices for tail-somite-standardized Procrustes landmark data.

	F1%	F5%	FB	H1%	H5%	HB	PS1%	PS5%	PSB
**F1%**	**0**	0.1396	0.0551	**0.0001**	**0.0001**	**<.0001**	0.03	0.6809	0.0017
**F5%**	0.009	**0**	0.0142	0.0046	0.0043	0.0116	0.0063	0.7811	0.0034
**FB**	0.0135	0.0163	**0**	0.0002	**0.0001**	**0.0013**	0.0209	0.1502	0.6304
**H1%**	0.011	0.0094	0.0163	**0**	**<.0001**	**<.0001**	**<.0001**	0.2033	**<.0001**
**H5%**	0.011	0.0094	0.0163	0	**0**	**<.0001**	**0.0003**	0.2101	**<.0001**
**HB**	0.011	0.0094	0.0163	0	0	**0**	**0.0008**	0.2547	**<.0001**
**PS1%**	0.0177	0.0224	0.0238	0.0246	0.0246	0.0246	**0**	0.2240	0.0623
**PS5%**	0.0089	0.0081	0.0167	0.0113	0.0113	0.0113	0.0172	**0**	0.3301
**PSB**	0.0183	0.0181	0.0117	0.0199	0.0199	0.0199	0.0257	0.0178	**0**

Procrustes distances below diagonal, P-values above. Significant distances after Bonferroni adjustment in bold (α = 0.0017). The three fixation groups (1% glutaraldehyde: 4% PFA, 5% glutaraldehyde: 4% PFA, Bouin's solution post-fixation) are designated 1%, 5% and B, respectively. Sequential treatment steps of embryo harvesting, fixation, and post-scanning are designated F, PS, and H, respectively.

**Table 2:** Mahalanobis distance and P-value matrices for tail-somite-standardized Procrustes landmark data.

	F1%	F5%	FB	H1%	H5%	HB	PS1%	PS5%	PSB
**F1%**	**0**	<.0001	<.0001	<.0001	<.0001	<.0001	<.0001	<.0001	<.0001
**F5%**	2.9902	**0**	<.0001	<.0001	<.0001	<.0001	<.0001	**0.2031**	<.0001
**FB**	4.3854	4.6631	**0**	<.0001	<.0001	<.0001	<.0001	<.0001	0.0044
**H1%**	2.8417	2.5339	4.5411	**0**	<.0001	<.0001	<.0001	<.0001	<.0001
**H5%**	2.8417	2.5339	4.5411	0	**0**	<.0001	<.0001	<.0001	<.0001
**HB**	2.8417	2.5339	4.5411	0	0	**0**	<.0001	<.0001	<.0001
**PS1%**	4.5411	5.4378	5.6312	5.5026	5.5026	5.5026	**0**	<.0001	<.0001
**PS5%**	3.3045	2.3739	5.0075	3.1154	3.1154	3.1154	4.4685	**0**	<.0001
**PSB**	5.2962	5.5633	4.0946	5.9924	5.9924	5.9924	5.9346	5.5924	**0**

Mahalanobis distances below diagonal, P-values above. Non-significant distances in bold.

**Figure 8:**
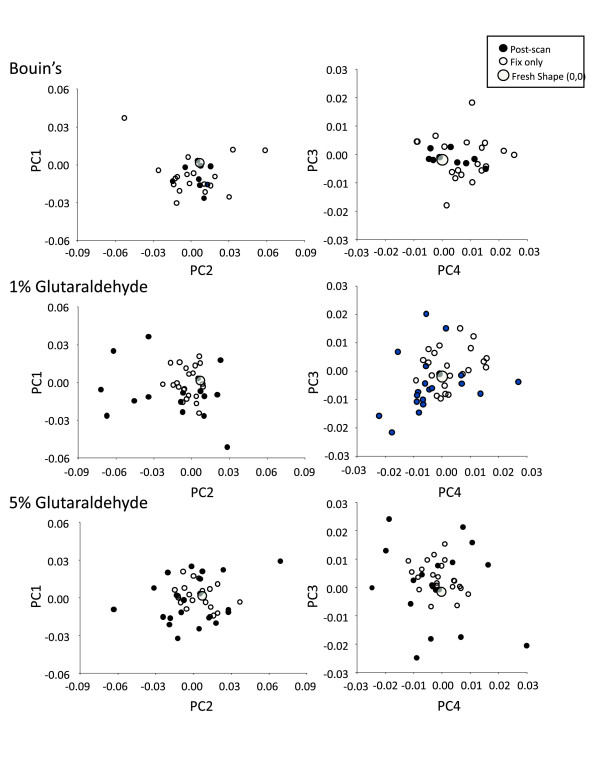
**PCA results showing the scatter of residual Procrustes distance values after normalization onto pooled-harvest sample shape data**. The scatters show the shape due solely to processing related distortion. The large grey circles show the harvest value, which has been normalized at (0,0) for each individual within each experimental group.

Figure 8 depicts PCA results showing the scatter of residual Procrustes distance values after normalization onto pooled-harvest sample shape data. Again, here the scatters show the shape due solely to processing-related distortion. The trends exhibited with the Bouin's experimental group contrast with both glutaraldehyde experimental groups. In the former, the net effect of μCT scanning was to reduce the amount of shape distortion associated with Bouin's fixation, as the scatter representing scanning effects tend to a tighter pattern around the harvest mean (0,0). In both the 1% and 5% glutaraldehyde experimental groups, shape distortion accumulated as the workflow progresses from harvesting to fixation to μCT scanning, as the scatters tend to form a diffuse pattern about the harvest mean shape (0,0).

## Discussion

Generally speaking, the particular choice of fixative depends on which particular histological feature a researcher wishes to demonstrate [23,24,26]. In the field of developmental biology, it is often the localization or activity level of a chemical factor synthesized within a particular differentiating cell type that is of interest. However, any gross morphological alterations induced by fixation are usually not of primary concern. Our goal was to measure the artifactual changes to craniofacial size and shape induced by fixation and μCT scanning.

When comparing μCT scans of Bouin's-fixed embryos to those fixed with either glutaraldehyde solution, the latter appeared larger and with smoother ectodermal surface texture. Formaldehyde-based fixatives (e.g., Bouin's solution and our glutaraldehyde mixtures) are known to cause either increases or decreases in tissue turgidity, sometimes causing swelling, while other times inducing shrinkage [23,24,26]. Despite the fact that the rendered μCT images represented in Figure 2 are to scale, they do not themselves support any precise explanation for the differences in size and texture associated with each fixative.

### Comparisons of Bouin's Solution and Glutaraldehyde fixation

In order to distinguish between scenarios of Bouin's solution-induced shrinkage versus glutaraldehyde-induced tumescence, initial craniofacial conditions were quantified and tracked through the course of our μCT scanning protocol. The most dramatic change in average centroid size was associated with using Bouin's solution secondary to 4% formaldehyde, decreasing average craniofacial centroid size by 26.5% (Figure 5A). Subsequent μCT scanning induced practically no further decreases in average craniofacial size. This pattern contrasts with our results using glutaraldehyde solutions (Figure 5B-C). Overall head size shrinkage was more limited in extent, reducing average centroid sizes in the end by 3.2% and 7.8% when using the 1% (Figure 5B) and 5% (Figure 5C) glutaraldehyde mixtures, respectively. Therefore, it appeared that each of the fixation procedures induced some degree of tissue shrinkage. A simple interpretation of the results is that our use of Bouin's solution initially dehydrated and shrunk the embryos to a greater extent prior to scanning, whereas aldehyde fixation was sufficient to maintain craniofacial size close to but just under original (harvest) centroid size values post-fixation and post-scanning, despite alterations to tissue composition and structure. This interpretation is consistent with our initial analysis of unstandardized data. Regressing Procrustes distance values against centroid sizes of individuals at each processing step within experimental groups (Figure 4B) shows that Bouin's has a more discernable effect on the scaling relationship.

Figure 6 compares the magnitudes of craniofacial shape deformations associated with sample processing steps within each of the three experimental groups. For the Bouin's fixation group, the most substantial shift in Procrustes distance was associated with fixation (Figure 6A), the same treatment step that induced the most significant change in average centroid size (Figure 5A). For both of the glutaraldehyde fixation groups, we were able to show that alterations in craniofacial shape appear to accumulate through the work flow, as Procrustes distances increase between each treatment step, being more substantial in association with scanning than with fixation. Unlike our findings with Bouin's fixation, this pattern of shape distortion is not as tightly associated with changes in average centroid size, as these latter values are fairly stable during the work flow, decreasing by only a fraction of that observed with Bouin's fixation.

To better understand how these observed shape distortions compare across experimental groups and processing steps, we tabulated a matrix of tail-somite stage-standardized Procrustes distances, further standardized to the mean landmark configuration for all unfixed, freshly harvested embryos (Table 1). UPGMA cluster analysis was applied to the matrix, yielding a phenogram (Figure 7) wherein the proximity of the terminal branches to the harvest mean represents the relative severity of artifactual noise experienced by each experimental group during the processing steps of fixation and scanning. According to the UPGMA analysis, embryos fixed with 4% formaldehyde + 5% glutaraldehyde experienced the least amount of craniofacial shape distortion. Any subsequent shape distortions associated with scanning, which are implied by Figure 6C, were apparently not enough to overcome the nesting of fixed with post-scanned embryos of the 5% glutaraldehyde experimental group. This was not the case for fixed and post-scanned embryos of the 1% glutaraldehyde group. While embryos fixed with 1% glutaraldehyde solutions nested just basal to fixed and post-scanned conditions of the 5% glutaraldehyde group, post-scanned embryos of the 1% glutaraldehyde fixation group are the most deviated in terms of treatment induced variation of any treatment step between or within groups. Embryos fixed with 4% formaldehyde and Bouin's solution nest together with the same embryos once μCT scanned. Similar to the 5% glutaraldehyde experimental group, any further changes in Procrustes distances associated with scanning implied by Figure 6A were not enough to overcome the similarities in standardized landmark configurations despite treatment induced shape deformations.

Taken together, the data suggest the following scenario. When using Bouin's solution to fix embryos preparatory to μCT scanning, distortions in overall shape are coupled to embryo shrinkage mainly during fixation. The degree of specimen dehydration and the rigidity that this confers embryos are adequate to stabilize the embryos during the time they are exposed directly to the air, while inside the μCT scanning device. Fixation with 4% formaldehyde + 1% glutaraldehyde induces a similar level of artifactual shape distortion as with 4% formaldehyde + 5% glutaraldehyde fixation and subsequent scanning, but did not appear to confer a similar level of shape stability. The large level of distortion in this case cannot be attributed to further degrees of specimen shrinkage during scanning and air exposure, since average centroid size was not very affected between fixation and post-scanning (Figures 4, 5B). This would imply that the source of distortion was perhaps physical deformation owing not to further dehydration, but rather to subtle gravitational collapse prior to taking the final photographs in lateral aspect (i.e., probably just before μCT scanning).

## Conclusion

Fixation and μCT scanning impart tissue distortions measurable in terms of organismal size and morphometric shape. In particular, Bouin's fixation, following primary fixation in 4% formaldehyde, induces a much greater degree of specimen shrinkage and shape distortion than fixation with 4% formaldehyde + 5% glutaraldehyde. In both cases, the shape variability is fairly stable throughout the process of data acquisition. Fixation with 4% formaldehyde + 1% glutaraldehyde, though adequate to resist changes in overall size, does not provide sufficient protection from randomly occurring shape distortions associated with scanning. This downfall may be mitigated if μCT scanning was performed while the specimen is immersed in a fluid environment rather than air. This option would require that the supporting liquid have a substantially lower radiodensity than the specimen (such as ethanol or methanol) [17,18]. However, this would entail substantial dehydration of the embryo and attending distortions in terms of tissue size and morphometric shape.

**Figure 9:**
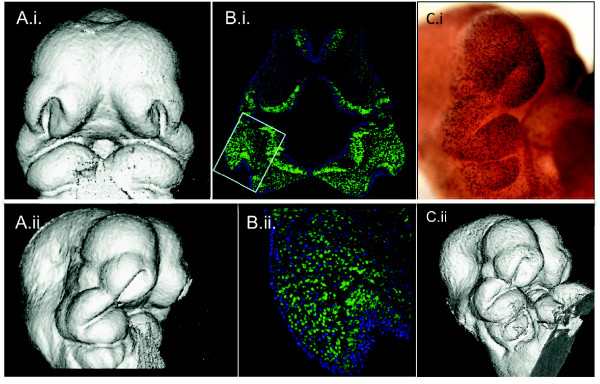
**μCT renderings and cell proliferation data from the same specimens**. **A**, 3D reconstruction of μCT taken after processing but before sectioning shown in anterior and right lateral 3/4 views. **B. i, ii **Hoescht 33342 staining to visualize cell nuclei (blue) with cells in S-phase visualized using EdU **+ **Alexa Fluor^® ^488 labeling (green) in frontal sections at the level of the maxillary prominence. **B.i **at 50× and **B.ii **and 200×. Small box in **B.i**. shows the region magnified below. **C**.ii, μCT rendering and same specimen (**C.i**) processed wholemount for anti-PHH3 primary antibody to identify M-phase cells shown in right lateral 3/4 view.

Coupling 3D morphological analyses with histological datasets is viewed as a crucial source of information with much potential to enrich understanding of morphogenetic mechanisms underlying organ growth and development [1-5,7-10,16]. For example, our research group is exploring methods to combine the analysis of regional variation in cell proliferation rates and micro-CT based morphometric data [16]. Such analyses possess the potential to reveal the relationship between cell proliferation data and morphometric variation within samples and can be used to compare this relationship among genotypes or groups that differ in characteristics of interest. We are employing multiple approaches in the attempt to visualize the 3D distribution of proliferating cells within craniofacial primordia (Figure 9). These approaches involve the development of computer-based methods for morphing multiple individuals to a mean shape, superimposing histological and computed microtomography data, averaging multiple individuals for such datasets to construct genotype or other group means, and the development of statistical methods to compare the distribution of immunohistochemical markers or regions of gene expression among groups [27]. Our goal of uniting μCT volumetric data pertaining to embryonic craniofacial size and shape with molecular expression data at histological resolutions will help us to understand how variation in basic morphogenetic processes shape and organize variation at the gross anatomical level. The ability to correlate morphological and molecular data at the individual embryo level will offer a new toolkit to elucidate the relationships between genotypic and phenotypic variation in the contexts of developmental and evolutionary biology as well as in clinical settings.

## Methods

### Sample

Embryos (n = 45) are of the C57BL/J strain. Dams were sacrificed using cervical dislocation 10.5 days post conception as indicated by the presence of a vaginal plug. Uteri were removed and placed into ice cold Liebovitz's L-15 Medium (Gibco^®^), wherein embryos were dissected and cleared of extraembryonic membranes. Hearts were dissected immediately so as not to obscure facial morphology in subsequent photography and μCT scans. To mitigate intra- and inter-litter developmental variability, embryos were staged according to the number of somites formed caudal to the hindlimb bud (tail-somites), providing a comparative measure of developmental age at a finer scale than is recorded by chronological age. To avoid inter-litter effects on patterns of size and shape variability, individuals from each litter were separated and assigned to one of three experimental cohorts differing in the way embryos were fixed after initial harvesting. Embryos were not sexed. Animal handling protocols were approved by the Animal Care Committee at the University of Calgary, and the mice were housed and cared for in accordance with the regulations of the Canadian Council for Animal Care.

### 2D digital photography

**Figure 10:**
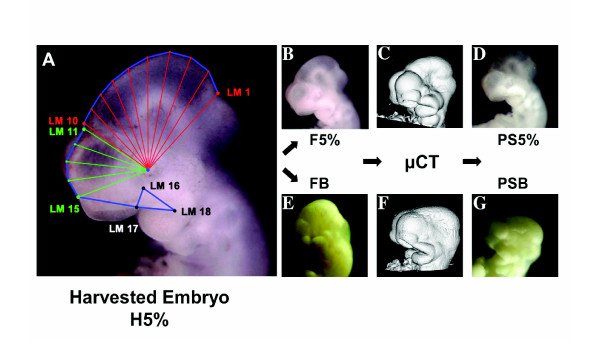
**A-D, photographic series of a single embryo from the 5% glutaraldehyde experimental group representing general workflow repeated for all individuals in sample**. **A**, freshly harvested, unfixed specimen representing initial photographs overlain with landmarking scheme: red fan samples midbrain, green fan samples left telencephalon. Wireframe outline of landmarks in blue. **B**, same embryo fixed with 4% formaldehyde + 5% glutaraldehyde. **C**, 3D μCT scan. **D**, final digital photograph of series taken after μCT scanning. **E-G**, imaging series of an individual representing workflow for individuals from Bouin's experimental group. **E**, Bouin's fixation. **F**, μCT scan. **G**, final photo of same specimen post μCT scan. Photographic series representing individuals of 4% formaldehyde + 1% glutaraldehyde experimental group not shown. **A**, **B**, **D**, **E**, and **G **at same magnification. **H5%**, harvested embryo of 5% glutaraldehyde experimental group. **F5%**, fixed embryo of 5% glutaraldehyde experimental group. **PS5%**, post-scanned embryo of 5% glutaraldehyde experimental group. **FB**, fixed embryo of Bouin's experimental group. **PSB**, post-scanned embryo of Bouin's experimental group. All embryos are shown in left lateral view.

For each embryo, a series of digital photographs was taken documenting external gross morphology at each processing step: Initial embryo harvest, fixation, and after μCT scanning. Immediately following uterine extraction, the freshly harvested embryos were positioned on their right sides in a flat-bottomed dissecting dish filled with ice cold Liebovitz's L-15 Medium and photographed using a dissection microscope (32×) to capture left lateral views of the cranium (Figure 10A). Due to their irregular shapes, the embryos did not lie completely flat on their right side, but rather tended to be tilted so that the underlying right forebrain and nasal process were slightly visible beneath those of the left (Figures 10A-G). We chose to photograph the embryos in this resting position because it provided consistent positioning with minimal manipulation. We assume that while this positioning produces rotational error, that error is consistent. Subsequent photographs were taken of each embryo following fixation and again following μCT scanning (Figures 10D and 10G). For these latter photographs, the same standards were followed except that embryos were placed in a dissection dish filled with ice cold phosphate buffered saline (PBS). Following [33], we assume in this analysis that rotational error is present in our shape data and has two components. One should be random and therefore would not be expected to introduce any systematic biases. On the other hand, it is reasonable to expect that with fixation- and scan-induced changes to embryonic shape would come systematic effects on embryo positioning during photography. This component would be represented in the linear combinations comprising each principal component describing shape variation (Figures 3 and 7) but is not explicitly isolated and accounted for. It is considered to be a source of statistical nuisance that must be recognized as an inherent but difficult to avoid methodological shortcoming. Interpretations of our data should be qualified with respect to this deficiency.

### Fixation

We divided our sample into three separate experimental groups. n = 18 embryos were fixed in a solution of 4% formaldehyde + 5% biological grade glutaraldehyde (5% glutaraldehyde experimental group) in PBS overnight at 4°C prior to scanning (Figure 1B-D). n = 19 embryos were similarly fixed in 4% formaldehyde + 1% glutaraldehyde (1% glutaraldehyde experimental group). n = 8 embryos were initially fixed at 4°C overnight in 4% formaldehyde before being secondarily fixed in Bouin's solution for 3.5 hours at room temperature (Bouin's experimental group) (Figure 1E-F). 3.5 hours of secondary fixation was an empirically determined minimum sufficient to provide consistent μCT scanning results for the Bouin's experimental group. Formaldehyde solutions were prepared from crystalline paraformaldehyde dissolved in PBS.

### Scanning Protocol

All embryos were imaged by μCT scanner (Skyscan 1072, Kontich, Belgium) at a 6.25 μm nominal resolution (42 kV, 98 μA, 0.90° rotation step, 2 frame averaging and a 3.8 s exposure time) and corrected for both flat field and random movement errors. Prior to scanning, embryos fixed in formaldehyde-glutaraldehyde solutions were immersed in Cysto Conray^® ^II Iothalamate meglumine (tyco Healthcare, St. Louis) at room temperature for 1 hour. Embryos post-fixed with Bouin's solution were not immersed in contrast agent. Embryos were removed from their respective liquid media, gently blotted to remove excess fluid, and carefully mounted on pieces of polystyrene inserted in Bijou tubes. The scans were reconstructed (CONE_REC, v1.4.4.0, Skyscan) and cropped (IMAGEJ, 1.37 v, http://rsb.info.nih.gov/ij). A volumetric reconstruction for each embryo was obtained in ANALYZE3D http://www.mayo.edu/bir/ (Figures 10C, F, 2A-C).

### Landmarks

Seven landmarks and nine semi-landmarks [28,29] were chosen to reflect the external outlines of embryonic brain and morphology of the frontonasal process and maxillary prominence as viewed in profile (Figure 10A). The following landmarks (LM) were plotted and digitized using tpsDig [30]: midbrain-hindbrain juncture (LM 1); juncture between right forebrain and midbrain (LM 10); juncture between left forebrain and midbrain (LM 11); juncture of left medial nasal process and left forebrain (LM 15); point between left eye, left maxillary process, and external ventrum of forebrain (LM 16); juncture of left lateral nasal process and left maxillary process (LM 17); juncture of mandible and left maxillary process (LM 18) (Figure 10A).

Semi-landmarks were identified in IMP using MakeFan6 [31], centering the vertices of two separate fans on the lens of the left eye (Figure 10A). One fan sampled the midbrain, extending eight rays with equal angular displacements between LM 1 and LM 10. The second fan sampled the left telencephalon between LM 11 and LM 15, extending three rays with equal angular displacements between LM 11 and LM 15. Semi-landmarks (SLM) interposed between LM1 and LM 10 (Figure 1A, red fan) and between LM 11 and LM15 (Figure 10A, green fan) were digitized using tpsDig and designated SML 2-9 and SML 12-14, respectively.

To assess the repeatability of the landmarks, we performed three repeated trials of 10 embryos, divided into groups representing the three fixation methods. At each trial, each embryo was positioned imaged and landmarked and the trials were conducted on different days. Since we use Procrustes superimposed landmarks in the analysis, we quantified measurement error as the variation in landmark position produced by measurement error as a proportion of the total variation in the measurement error sample. Shape is dimensionless and so this is the appropriate way to quantify measurement error in such data. The percentage measurement error can be minimized artificially by conducting the trials on very dissimilar specimens (e.g. varying greatly in stage). This inflates the among-individual variance and so makes the measurement error variance appear smaller. For this reason, the measurement error trial was conducted on embryos from two litters with minimal variation in tail-somite stage. Our estimate of the percentage of variation accounted for by measurement error is thus conservative (large) compared to the actual percentage in the full analysis.

### Geometric morphometrics

Landmark and semi-landmark data were aligned using Semiland6 [31] using the criterion of minimum Procrustes distance. In Procrustes analysis, the configuration of landmarks corresponding to each individual is scaled to centroid size and then the whole sample is superimposed, removing differences in position and rotation, using a least-squares based method [32]. After Procrustes superimposition, the dataset can be analyzed in terms of shape and size variation.

#### Measuring Craniofacial Size

For each experimental group we calculated the average centroid size at each of the three processing steps: Initial embryo harvest (Figure 10A), followed by fixation (Figure 10B and 10E), and post-μCT scanning (Figure 10D and 10G). The effect of fixation and scanning on embryonic size was measured as the ratio of averaged centroid sizes between tissue processing steps within experimental groups.

#### Measuring Craniofacial Shape

We performed two initial analyses on our 2D morphometric dataset prior to standardizing it to tail-somite stage 16. Principal Components Analysis (PCA) was performed on the "raw," unstandardized dataset (Figure 4A). Secondly, we regressed Procrustes distance values against craniofacial (centroid) sizes (Figure 4B).

An important advantage of Procrustes-based methods is the ability to quantify and correct for covariates such as age, weight, and somite stage. Embryo shape and size changes dramatically with ontogeny and can vary significantly even with litters. Ontogenetic shape variation, therefore, can thus be quantified and removed as shown in previous work [13,15,16,33]. To do this, Procrustes coordinates are regressed on tail-somite number for embryos using the pooled within-sample multivariate regression of tail-somite number on shape in MorphoJ [34] or a pooled multivariate regression of tail-somite stage on shape in IMP [26] as described in [29]. The residuals of these regressions are then used to create a dataset standardized to a particular tail-somite stage using 3Dstand [31]. We standardized the entire dataset to tail-somite stage 16 for analysis.

To determine how fixation and scanning influence shape, we removed the among-individual variation in shape from the stage-standardized data. This was done first by normalizing each individual landmark configuration to the mean shape of freshly harvested, unfixed embryos from each of the three experimental groups and secondly by representing each individual in terms of the shape deviation produced by fixation and scanning. Thus, we created a shape dataset in which only the "artifact" variation produced by fixation and scanning was present.

To compare the magnitudes of the distortions produced by different treatments (i.e., harvesting, fixation, scanning), we calculated the Procrustes shape distance [20] between each embryo and itself after each treatment (Figure 5A-C). This metric is a relative measure of the amount of shape distortion within experimental groups produced by the treatment. To compare the shape variation between and within experimental groups, we performed PCA on the shape dataset (Figure 7), and calculated the permutation tests for both the Procrustes (Table 1) and Mahalanobis (Table 2) distances between experimental groups (10,000 permutations). Unweighted Pair Grouping using Mathematical Averaging (UPGMA) was applied to the matrix of Procrustes distances to compress and express the data in terms of a dendrogram (Figure 6).

### Cell proliferation

#### EdU labelling, immunohistochemistry, tissue sectioning

Proliferating cells were demonstrated by labelling replicating DNA with EdU [35] using the Click-iT™ EdU Alexa Fluor^® ^488 Imaging Kit (Invitrogen). Dams were injected with 200 μl of EdU in PBS (1 μg/μl) 30 minutes prior to embryo harvesting and overnight fixation with either Bouin's solution or formaledehyde + glutaraldehyde mixtures at 4.0°C. Following washes in PBS, embryos were secondarily fixed in methanol: DMSO (4:1) overnight at -20.0°C. Embryos were again washed in 0.03% Triton in PBS for several hours at room temperature before application of azide reaction cocktail per manufacturer's instructions. Wholemount embryos were then washed for 15 minutes in 1 μg/μl Hoescht 33342 in PBS to label genomic DNA. The specimens were then embedded in plastic resin and sectioned at 10 μm. Alternatively, embryos were fixed in 4% formaldehyde overnight at 4.0°C and processed for αPHH3 immunohistochemistry using standard protocols.

## Authors' contributions

ES, TEP, HAJ, CCL, and BH conceived of the study. TEP, ES, and BH performed the statistical analyses. ES, TEP, HAJ, CT, and JG participated in embryo μCT scanning and 2D landmarking. ES, JG, CCL, and JCB performed histochemical preparations. BH and CWS supervise the team working on the larger project of which this paper is a component. All authors approved the draft and declare no competing interests. Lab website http://homepages.ucalgary.ca/%7Emorpho/index.html.
